# Caught in the Act: Variation in plastid genome inverted repeat expansion within and between populations of *Medicago minima*

**DOI:** 10.1002/ece3.6839

**Published:** 2020-09-29

**Authors:** In‐Su Choi, Robert Jansen, Tracey Ruhlman

**Affiliations:** ^1^ Department of Integrative Biology The University of Texas at Austin Austin Texas USA; ^2^ Centre of Excellence in Bionanoscience Research Department of Biological Sciences Faculty of Science King Abdulaziz University Jeddah Saudi Arabia

**Keywords:** alfalfa, gene conversion, homologous recombination, inverted repeat expansion, medicago, plastid haplotype variation, plastome evolution

## Abstract

The inverted repeat (IR) lacking clade (IRLC) is a monophyletic group within the Papilionoideae subfamily of Fabaceae where plastid genomes (plastomes) do not contain the large IR typical of land plants. Recently, an IRLC legume, *Medicago minima*, was found to have regrown a ~9 kb IR that contained a number of canonical IR genes, and closely related *M. lupulina* contained an incomplete IR of ~425 bp. Complete plastomes were generated for seven additional species, putative members of the *M. minima* clade. Polymerase chain reaction was employed to investigate the presence of the IR across *M. minima* and *M. lupulina* including individuals of nine and eight Eurasian and North African accessions and 15 and 14 Texas populations, respectively. While no sequence similar to the ~9 kb IR was detected among the seven newly sequenced plastomes, all Eurasian and North African accessions of *M. minima* contained the IR. Variation in IR extent was detected within and between the Texas populations. Expansions of 13 bp and 11 bp occurred at the boundaries of both IR/small single‐copy regions, and populations had one or the other expansion, but not both. Expansion of the IR was not detected in the accessions from Eurasia and North Africa suggesting recent mutations yielded at least two additional plastid haplotypes in *M. minima*.

## Graphical abstract

A recent study revealed the reemergence of the plastome inverted repeat (IR) in *Medicago minima*, which belongs to a clade of legumes defined by its absence. Seven putatively related plastomes were sequenced, and accessions/populations of *M. minima* from Europe, Asia, Africa, and Texas were assayed for presence of the IR and variation in its boundaries. While “Old World” populations of *M. minima* all contained identical IRs to the accession that was previously sequenced, variation in the IR boundaries was detected within and between Texas field‐collected populations. Range expansion, haplotype distribution, and IR expansion mechanisms are discussed.

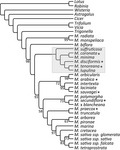

## 1 INTRODUCTION

A plastid genome (plastome) containing large and small single‐copy regions (LSC and SSC, respectively) separated by a large inverted repeat (IR) is widely acknowledged to represent the ancestral form of the plastome monomer of land plants. The typical plastome IR is on the order of 25–30 kb with most size variation driven by expansion and contraction of IR boundaries (Jansen & Ruhlman, 2012; Ruhlman & Jansen, 2014). The ancestral form is not, however, ubiquitous among photosynthetic plants. In disparate lineages, the IR has massively expanded, contracted, and been entirely lost (Ruhlman & Jansen, 2018; Zhu et al., 2016).

Major IR expansions identified among *Pelargonium* species yielded the largest plastomes, which contain IRs that ranged up to ~88 kb (Weng et al., 2017). Likewise, some of the smallest plastomes among autotrophic plants lack the IR entirely (Blazier et al., 2016; Ruhlman et al., 2017; Sabir et al., 2014; Sanderson et al., 2015), or contain highly reduced remnants of the ancestral repeat (Guisinger et al., 2011). In at least two cases, IR reemergence has been documented within lineages where the IR was lost. The highly unusual long‐branch clade (LBC) species of *Erodium* exhibit novel IR structures ranging from 25 kb to more than 47 kb concomitant with plastome expansion (Blazier et al., 2016). While it is somewhat obscure whether there were two independent IR losses, or a shared loss with reemergence exclusively in the LBC for the return of the *Erodium* IR, the case in one clade of legumes is clear.

Loss of the IR is the predominant character that defines the IR lacking clade (IRLC; Wojciechowski et al., 2004) of papilionoid legumes. Since the earliest example of IR loss was uncovered (Kolodner & Tewari, 1979), direct DNA sequencing and more recently next generation, high‐throughput sequencing have facilitated analyses that supported the monophyly of the IRLC (McMahon & Sanderson, 2006; Schwarz et al., 2017; Wojciechowski et al., 2000). The genus *Medicago* contains about 87 described species (Small, 2011) and belongs to the tribe Trifolieae, which is nested within the IRLC (Cardoso et al., 2015; The Legume Phylogeny Working Group, 2017). The most well recognized *Medicago* species is the food and forage crop alfalfa (*M. sativa*. ssp. s*ativa*). Also notable is the plant research model *M. truncatula*, with high reproductive rates and amenability to genetic manipulation. *Medicago* were not considered highly rearranged (Cai et al., 2008; Sveinsson & Cronk, 2016); however, this assumption was based on a single plastome, that of *M. truncatula*. Expanded sampling included 19 species representing all the major clades in the genus and found only modest divergence among congeners with regard to structural organization overall (Choi et al., 2019). However, one clade contained unique variation in repeat structure including an incomplete IR in *M. lupulina* and the presence of a novel IR of ~9 kb in *M. minima*. The sequences that flank the novel IR differ from the canonical boundary sequences (Mower & Vickrey, 2018; Yamada, 1991), yet the IR of *M. minima* contains a portion of the ribosomal operon (*rrn23* … *trnN*‐GUU), with the remaining IR core genes (*trnV*‐GAC…*trnA*‐UGC) and other common IR sequences lying upstream of IRb (Choi et al., 2019).

The previous study included single individuals for just three taxa in the *M. minima* clade. To examine the evolution of IR reappearance in the clade, plastomes for seven taxa suggested to be close relatives in phylogenetic studies (see Small, 2011) were completed. Additionally, populations of *M. minima* and *M. lupulina* from across the native range, Northern Africa, and Central Asia, as well as field‐collected populations from across Texas were assayed for the presence of the novel IR and variation in its extent. Questions about the role of the IR in plastome recombination and replication have persisted since its discovery. Investigating variation in IR presence and extent within and between populations and closely related species may illuminate our understanding of the role of the IR and the mechanisms of plastome maintenance.

## 2 MATERIALS AND METHODS

### 2.1 Additional taxon selection and plastome sequencing

Phylogenetic studies suggested that additional taxa could be included in the monophyletic group that includes *M. minima* that were not included in the previous analyses (Small, 2011). Accessions of seven taxa were acquired from USDA‐GRIN (U.S. Department of Agriculture Germplasm Resources Information Network) (Table S1). Seeds were germinated and grown in the greenhouse at UT Austin and emergent leaves were collected in liquid nitrogen from single individuals. All DNA protocols followed Choi et al. (2019), including isolation, sequencing, assembly, and annotation of plastomes. Completed plastome sequences were submitted to NCBI and GenBank accession numbers are given in Table S1. Repeat content of newly sequenced plastomes was estimated according to Choi et al., 2019. Gene sequences were extracted from the new taxa and combined with the phylogenetic data set from the previous analysis (Choi et al., 2019). All shared protein‐coding sequences (69; see Table S2, (Choi et al., 2019) for 27 *Medicago,* and eight outgroups were used to infer relationships. All phylogenetic methods followed Choi et al. (2019).

### 2.2 PCR screen of *Medicago minima* and *M. lupulina* accessions

Nine accessions of *Medicago minima* and eight of *M. lupulina* were acquired from USDA‐GRIN (Table S2) to represent populations from Asia, Europe (Eurasia), and North Africa. Seeds were germinated and grown in the UT greenhouse. Emergent leaves were collected in liquid nitrogen, and DNA was isolated using the NucleoSpin Plant II (Macherey–Nagel, Düren, Germany). Texas field collections included 15 populations of *M. minima* and 14 of *M. lupulina*. Young leaves were harvested from a minimum of 10 individuals in each population and frozen in liquid nitrogen for DNA isolation. See Table S3 for accessions, location, and voucher information.

Individuals representing all included accessions, both from USDA and field collected, were vouchered and deposited in the TEX‐LL.

A PCR screen was applied to all accessions and employed primers utilized by Choi et al. (2019) to amplify IR junction sites in *M. minima* and to assess a small inverted repeat of interest identified previously in *M. lupulina* (Choi et al., 2019). Initial screening of USDA accessions and Texas populations of *M. minima* included three individuals, while single individuals were screened from each accession of *M. lupulina*. Four reactions were conducted for *M. minima* to amplify IR boundary regions (boundaries of IR_A_ with large and small single‐copy sequences are abbreviated as *J*_LA_ and *J*_SA_, respectively; the boundaries for IR_B_ follow the same convention, that is, *J*_LB_ and *J*_SB_) and two reactions amplified the repeat region in *M. lupulina*. Subsequent reactions amplified two polymorphic sites detected in *M. minima* (*J*_SA_, *J*_SB_) and were conducted with additional seven individuals from each Texas population (Tables S2 and S3). Sanger sequencing of all amplicons was carried out at the University of Texas Genomic Sequencing and Analysis Facility. Amplicon sequences were aligned to corresponding plastome loci in complete plastomes of *M. minima* (MK460499) and *M. lupulina* (MK460497) with MAFFT v.7.017 (Katoh & Standley, 2013), implemented in Geneious, using the default settings. Genbank accession numbers for sequenced amplification products are provided in Table S4.

## 3 RESULTS

### 3.1 Sequencing and phylogenetic inference for seven new *Medicago* plastomes

**Figure 1:**
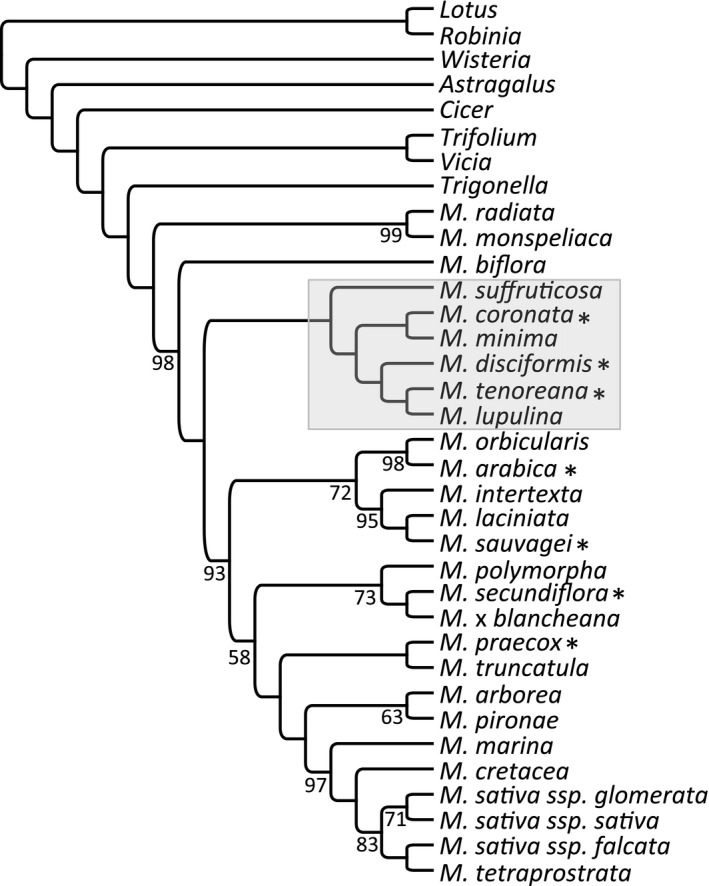
Relationships among 27 *Medicago*. The cladogram depicting relationships of *Medicago* (M) and outgroups was based on maximum likelihood phylogenetic inference using 69 protein‐coding genes shared across all taxa. Bootstrap values less than 100 are shown at the nodes. Newly sequenced plastomes are indicated by asterisks, and the *M. minima* clade is boxed in gray. ssp., subspecies

Complete plastomes were assembled for seven *Medicago*. Sequencing statistics and overall plastome characteristics are reported in Table 1, while Table S1 contains USDA‐GRIN, NCBI, and voucher accession numbers. Phylogenetic inference based on 69 shared plastid genes across 27 species placed three of the seven new *Medicago* in the *M. minima* clade. *Medicago tenoreana* and *M. lupulina* formed a clade sister to *M. disciformis*. These three together were sister to a clade that included *M. coronata* with *M. minima*. The other four taxa, *M. arabica*, *M. sauvagei*, *M. secundiflora,* and *M. praecox* were distributed within a large clade that was sister to the *M. minima* clade (Figure 1). None of the newly sequenced plastomes contained a structure resembling the IR of *M. minima*. The inclusion of *M. secundiflora* extended the size range of *Medicago* IR lacking plastomes, reported here as ~120 to ~126 kb (Table 1; Choi et al., 2019).

**Table 1:** Plastome sequencing statistics for seven *Medicago*

Taxon	Reads	Unit length (bp)	Plastome coverage	GC (%)	Repeat content (%)
*M. praecox*	48,984,174	122,724	6,743	34.0	4.27
*M. secundiflora*	48,641,318	120,166	4,920	34.7	5.06
*M. sauvagei*	44,530,096	124,743	5,239	33.9	4.65
*M. arabica*	44,443,436	125,028	5,081	34.4	5.72
*M. disciformis*	49,327,710	121,039	5,768	34.2	4.46
*M. coronata*	49,364,864	123,864	5,784	34.2	5.99
*M. tenoreana*	47,121,870	120,857	8,310	34.2	5.74

### 3.2 PCR screening for IR distribution and extent

Single individuals from USDA accessions and accessions collected from field sites in Texas were screened for variable sites of interest. For *M. lupulina*, PCR primers were designed to amplify repeat regions with possible analogy to the *M. minima* IR (Choi et al., 2019). Each of the two repeat units (aligned length two copies, 425 bp) were amplified for each individual. Sequenced amplicons from 22 M*. lupulina* individuals (eight from USDA; 14 from Texas; Tables S2 and S3) revealed no variation in the length or of boundary positions of the repeats relative to the original accession sequenced by Choi et al. (2019).

**Figure 2:**
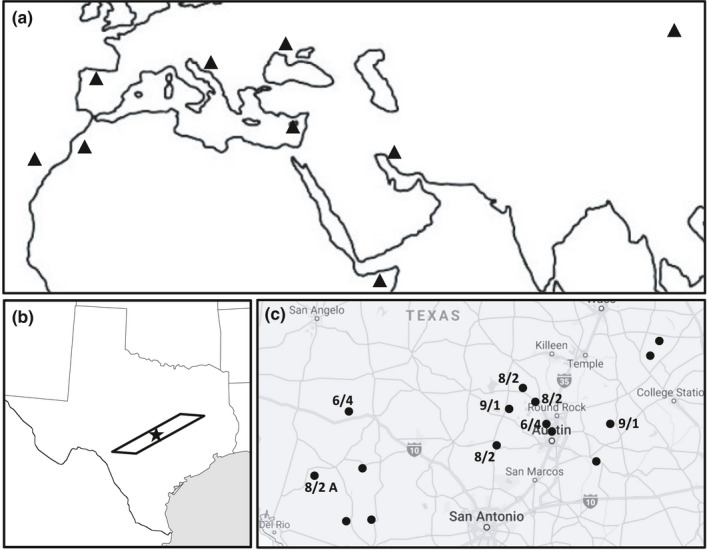
Accession origins of *Medicago minima*. Accessions of *M. minima* were acquired from USDA GRIN and collected from field sites in Texas. (a) Triangles indicate approximate origin of accessions from Eurasia and northern Africa (Table S2). An overview of transect (polygon) that included collections sites for all Texas accessions (b) along with specific locations, including haplotype distribution (c). Black star in (b) indicates the location of the state capitol, Austin TX. In (c), dots indicate individual sites. The values at each site give the O haplotype/B haplotype, except for one site (8/2 A), which indicates O/A haplotype distribution. Populations where only the O haplotype was detected lack an associated value (Table S3)

In all, 177 individuals of *M. minima* were screened for variation in IR/SC boundaries. Initial screens included three individuals of the ten sampled from each population. Accessions acquired from USDA, representing Eurasian and North African populations (Figure 2, Table S2), all contained the ~9 kb IR identified in the original sequenced plastome (Choi et al., 2019) and were identical with respect to the IR/SC boundaries. While no variation was detected in the position of the IR/LSC junction, initial screens of the Texas populations did reveal variation in the junction of the IR and the SSC region (Table S3). The remaining seven individuals from each Texas population were screened using primers to amplify the variable junctions at both IR_A_/SSC (*J*_SA_) and IR_B_/SSC (*J*_SB_).

**Figure 3:**
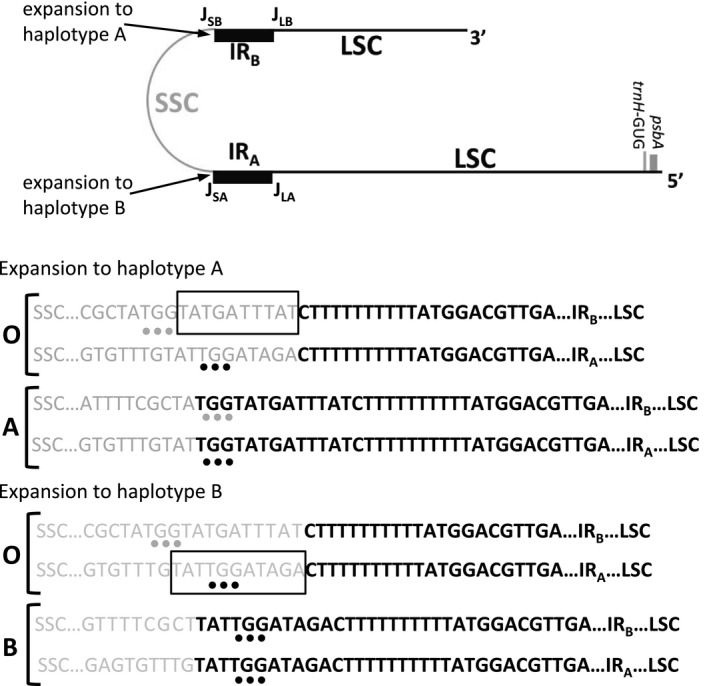
Inverted repeat length variation in *Medicago minima*. Both polymorphisms extended the IR into the small single‐copy region (SSC). A schematic representing a single unit of the *M. minima* plastome is presented for context. Inverted repeat expansion to the **A haplotype** (**A**) included an additional 13 bp of single‐copy sequence (gray) in the IR. The expansion includes 10 bp upstream of J_SB_ (boxed) that were single copy in *M. minima* plastomes that lack the expansion (**O**). Five base pairs were inserted at J_SA_ (TTTAT), and five base pairs of formerly single‐copy sequence have likely undergone gene conversion (**ATA**GA ➔ **TAT**GA) homogenizing the two repeats. The extension of the IR places both junctions adjacent to an existing three‐base sequence (TGG, gray, and black dots) that is present in near both ends of the SSC in *M. minima* plastomes that contain unexpanded IRs. The polymorphic expansion yielding **haplotype B** (**B**) included 11 bp in the *M. minima* IR relative to the unexpanded **O haplotype.** Gene conversion templates are boxed. bp; base pairs. LSC; large single‐copy region. SSC; small single‐copy region. IR; inverted repeat. J_SB_ and J_LB_; boundary of IR_B_ with SSC and LSC, respectively. J_SA_ and J_LA_; boundary of IRA with SSC and LSC, respectively

The sequences at *J*_SA_ and *J*_SB_ were polymorphic both within and between the Texas populations. Eight of the 15 sampled populations were polymorphic for expansions at either *J*_SA_ or *J*_SB_, but not for both yielding three plastome IR haplotypes: Type O, representing the unexpanded IR identified in the originally sequenced plastome of *M. minima* (Choi et al., 2019); type A, derived from expansion that initiated at *J*_SB_; and type B, derived from expansion that initiated at *J*_SA_ (Figure 3). Haplotype designation of “A” or “B” indicates the IR junction at which single‐copy sequence was overwritten resulting in expansion of the IR. Figure 2 and Table S3 summarize the geographic distribution of IR boundary variation among the Texas populations. While both polymorphisms extended the IR into the SSC, the less common was expansion to yield the A haplotype. This expansion, which was identified in a single population, appeared to include an additional 13 bp of single‐copy sequence in the IR. The expansion includes 10 bp upstream of *J*_SB_ that were single copy in *M. minima* plastomes that lack the expansion. Five base pairs were inserted at *J*_SA_ (TTTAT), and five base pairs of formerly single‐copy sequence have likely undergone gene conversion (**ATA**GA ➔ **TAT**GA) homogenizing the two repeats. Coincidentally, extension of the IR places both SSC junctions adjacent to an existing three‐base sequence (TGG) that is present near both ends of the SSC in *M. minima* plastomes that contain unexpanded IRs. This three‐base sequence does not appear to have been duplicated through IR expansion, but given its identical sequence at both junctions may be considered as included in the IR of the A haplotype. The polymorphic expansion yielding haplotype B included 11 bp in the *M. minima* IR relative to the unexpanded O haplotype. This expansion appeared less complex, with the sequence formerly found in the SSC adjacent to *J*_SB_ overwritten by gene conversion (Figure 3).

## 4 DISCUSSION

The phylogeny inferred in Choi et al. (2019) indicated that *M. minima* and *M. lupulina* were sister species, suggesting that repeat‐related phenomena were shared between them. The inclusion of three of the new plastomes in the *M. minima* clade parsed *M. minima* from *M. lupulina* (Figure 1). The lack of any structure that resembled the novel 9 kb IR or possible precursor sequences identified in the previous study (Choi et al., 2019) in the new taxa, together with the finding that *M. minima* and *M. lupulina* are not sister taxa support independence between the 9 kb IR of *M. minima* and the two repeat units of *M. lupulina* (aligned length two copies, 425 bp).

Within species variation in plastome sequence and structure has been reported for a few groups of angiosperms and is often associated with geographical isolation of breeding populations (Barnard‐Kubow et al., 2014), experimental crosses or among cultivars (Gurdon & Maliga, 2014). Less commonly reported however is plastome variation among individuals within natural populations. Plastome transmission is primarily uniparental and predominantly maternal in most groups providing for the assumption that plastomes will be uniform among individuals due to the lack of sexual recombination (Birky, 2001; Greiner et al., 2015). In biparental lineages, those that inherit their plastomes from both the pollen and seed parent, the potential for plastome haplotype diversity is greatly increased.

Several studies have investigated the mode of organelle inheritance in different species of *Medicago*. Many efforts have been dedicated to characterizing alfalfa (*Medicago sativa* ssp. *sativa*) and the closely allied taxa of the *M. sativa* complex, which contains both tetraploid (2*n* = 4*x* = 32) and diploid (2*n* = 2*x* = 16) genotypes (Quiros & Bauchan, 1988). The members of the complex appear to be interfertile, as hybrids have been achieved both within and between ploidy levels (Masoud et al., 1990; Quiros & Bauchan, 1988; Schumann & Hancock, 1989). Both the diploids and the tetraploids of the complex are allogamous and employ varying degrees of self‐incompatibility, lending to their value as agricultural crops as allogamy often results in larger individuals and increases amenability to hybrid production. Inheritance modes have been identified as predominantly uniparental paternal, biparental, and uniparental maternal, depending on a number of variables (Lee et al., 1988; Nagata et al., 1999; Schumann & Hancock, 1989; Smith, 1989; Zhu et al., 1993).

The diploid annuals of *Medicago* have been less investigated. These diminutive species have carried only passing interest as a source of germplasm to improve the crop species *M. sativa*, due in part to their recalcitrance to hybridization. The annual species are autogamous, with both male and female reproductive structures completely contained within the minute flowers. Only rare instances of hybridization have been reported, and only under experimental conditions, where abnormalities ranged from early embryo abortion to chlorophyll deficiencies in hybrid progeny (Quiros & Bauchan, 1988 and references therein). Among the diploid annuals, *M. truncatula* has received the most attention as a genetic model system. Mechanical cross‐pollination experiments designed to examine plastome inheritance have employed ecotypes that contain polymorphic sites so that the parental origin of the sequence marker can be followed in “hybrid” progeny (i.e., Matsushima et al., 2008). While both seed and pollen parental markers were detected in cotyledons of F1 progeny, leaves evaluated later in development tended to retain only one or the other marker. Populations of F2 progeny raised from the seed of a single mother uniformly showed the markers of either parent supporting the notion that vegetative segregation (sorting out) resolves rapidly in this system (Birky, 1978; Johnson & Palmer, 1989).

The annual diploid *Medicago minima* is a predominantly self‐pollinated inbreeder that yields highly uniform progeny and is naturalized the world over (Small, 2011). Although omitted from an 1891 list of *Medicago* reported in a flora of Western Texas (Coulter, 1891), *M. minima* was recorded in North America (Alabama and Louisiana) in a 1901 publication from the US Department of Agriculture (Mohr, 1901). A USDA publication from 1913 discusses *M. minima*, along with several other nonperennial *Medicago* that were introduced to study their agronomic value (McKee & Ricker, 1913). By 1914, a herbarium specimen of *M. minima* was collected in Travis County, Texas (M. S. Young s.n., TEX00340358, TEX‐LL). Later reports include mentions of the species in Virginia (Fernald, 1941), Oklahoma (Hopkins et al., 1943), Arizona, and California (Howell, 1949). Today, *M. minima* can be found on six continents and is particularly common in disturbed habitats (Small, 2011). Most likely, multiple introductions of *M. minima* throughout North and South America in association with livestock imports have contributed to the present day populations in the Americas. Its burr‐like appendages on the seed capsules allow attachment to livestock as well as wildlife further contributing to range expansion.

Among the diploid *Medicago*, *M. minima* has received more recent notice for its potential as a forage particularly in the arid regions of south‐central Texas (Muir et al., 2005; Smith et al., 2008; Ueckert et al., 2003; http://aggieclover.tamu.edu/files/2010/06/ForageLegumesTexas.pdf). Recognized by its common name, little burr medic, *M. minima* was suggested to have been introduced to Texas sometime in the early twentieth century (Diggs et al., 1999) in approximate agreement with the herbarium record from 1914. A number of ecotypes/cultivars were collected from several locations in Texas for evaluation of tolerance to the colder and/or drier regions of the state (Ocumpaugh, 2001) by researchers from Texas A&M University. One cultivar collected from a grass pasture near Devine, TX showed particular promise on the poor soils in the southwestern part of the state. The unimproved cultivar “Devine” was released in 2005 (Smith et al., 2008) and registered to Texas A&M AgriLife foundation seed (Ocumpaugh et al., 2007). Currently, little burr medic “Devine” is produced and distributed under license by Pogue Agri Partners of Kenedy, TX, and sold throughout Texas and Oklahoma.

Mixed plastome haplotypes were identified within and between Texas collected populations of *M. minima,* while a single haplotype was detected among 10 populations across Eurasia and northern Africa. All individuals from all accessions contained at minimum the ~9 kb IR identified by Choi et al. (2019). This suggests that events leading to the reemergence of the IR in *M. minima* initiated prior to range expansion in Eurasia and northern Africa, and subsequent introduction to the Americas. Further expansion of IR boundaries is more obscure with respect to timing. A quick search of the US National Germplasm System site delivers 365 accessions across Eurasia and northern Africa, with sampling that includes just 10 accessions of progenitor populations containing either expansion may have been missed. Alternatively, either of the two IR expansions detected in the Texas populations could have arisen since introduction of the species to the Americas, the definitive timing of which remains uncertain as there were likely multiple and unintentional introductions. Regardless, the detection of populations with variable IR structure exclusively in the Texas populations suggests the mutations are recent.

Whether the frequencies of the detected plastome variations will change in the Texas populations will likely be independent of the parameters affecting breeding populations as outcrossing is rare in *M. minima*. In a predominantly or entirely selfing species, mode of inheritance would be unlikely to influence organelle haplotype diversity as the two gametes are inherited from the same parent, increasing homozygosity and reducing haploid and organelle gene flow. Reports of biparental inheritance of organelle genomes in *Medicago* have relied on either outcrossing taxa of the *M. sativa* complex or artificially fertilized diploid taxa. The presence of plastid haplotype variation in the Texas populations was more likely a result of intentional or unintentional mixture of seeds from different source populations. All collections sites for this study were current or former ranch lands, or current recreational hunting sites, which are commonly seeded with forage mixtures of *Medicago* species.

The more commonly observed variant haplotype included an additional 11 bp in the IR, while the less common variant included 13 additional bases, both extending the IR into the SSC. Branch migration of a Holliday junction across either IR/SSC boundary can result in heteroduplex formation prior to stalling of the replication fork. Resolution of the heteroduplex restarts replication with copy correction (gene conversion) yielding the parental situation or migration of the IR boundary depending on the template sequence. In both variants, no IR adjacent gene was disrupted by the gene conversion that homogenizes the IR, allowing the expansion to be retained. This mechanism has been invoked to explain small migrations in IR/SC boundaries in many land plants (Goulding et al., 1996; Wang et al., 2008; Zhu et al., 2016).

Incorporation of single‐copy sequence by branch migration across IR boundaries is unlikely to significantly expand the novel ~9 kb IR in *M. minima*. The extent of noncoding sequence flanking the IR should limit small scale expansion caused by Holliday junction migration across the IR boundaries. There remain 4 bp and 39 bp of noncoding sequences flanking the LSC boundaries with IR_A_ and IR_B_, respectively, whereas 254 bp and 96 bp separate the IR_A_ and IR_B_ boundaries from SSC coding regions, respectively. These regions of noncoding sequence may be incorporated into the IR piecemeal; however, the need to maintain coding regions will require a mechanism capable to duplicate larger expanses of sequence. The recombination‐based mechanisms hypothesized to have played a role in reemergence of the IR in an IRLC legume could continue to include genes presently situated adjacent in the large (i.e., *rpl20*, *ycf1*) or small (i.e., *psbB*, *trnA*‐UGC) single‐copy regions. All of the genes contained the canonical IR are currently situated upstream of IRb in the SSC and remain in the same order those in typical IR containing plants.

Denser sampling in the *M. minima* clade failed to identify examples of IR expansion in closely related taxa, but instead revealed variation in the extent of the novel 9 kb IR across different naturalized populations of *M. minima* in Texas relative to germplasm accessions from Europe, Africa, and Asia. The presence of the IR in all accessions of *M. minima* along with the two unique IR boundary movements could suggest relaxed control of IR extent in species, if in fact such mechanisms exist. Small‐ scale movements of IR boundaries via gene conversion are thought to be random (Goulding et al., 1996) but are likely influenced by controls affecting homologous recombination.

Expanded sampling of *M. minima* populations throughout the Americas may help to elucidate source germplasm for the plastome haplotypes that were identified in central Texas, supporting a clearer chronology for the observed IR changes. Similar population level surveys focused on outcrossing taxa that display unusual IRs may provide a population genetics system to evaluate the differential fitness of plastome/IR haplotypes in a given environment. Such a study could illuminate long‐standing questions about the role of the plastome IR.

## CONFLICT OF INTEREST

None declared.

## AUTHOR CONTRIBUTIONS

**In‐Su Choi:** Conceptualization (equal); data curation (lead); formal analysis (lead); funding acquisition (equal); investigation (equal); methodology (equal); project administration (supporting); software (lead); supervision (equal); validation (equal); visualization (supporting); writing–original draft (supporting); writing–review and editing (supporting). **Robert Jansen:** Conceptualization (equal); data curation (supporting); formal analysis (supporting); funding acquisition (equal); investigation (equal); methodology (equal); project administration (equal); resources (equal); supervision (equal); writing–review and editing (supporting). **Tracey A. Ruhlman:** Conceptualization (equal); formal analysis (supporting); funding acquisition (equal); investigation (equal); methodology (equal); project administration (equal); resources (equal); supervision (equal); visualization (lead); writing–original draft (lead); writing–review and editing (lead).

## Supporting information

Appendix S1–S4

## Data Availability

DNA sequences were deposited in GenBank. GenBank and USDA‐GRIN accession numbers, sample voucher information, and collection locations are provided in Appendix S1.
