# Supplementary material for: Caught in the Act: Variation in plastid genome inverted repeat expansion within and between populations of Medicago minima
Source: Ecol Evol. 2020 Sep 29;10(21):12129–37. doi: 10.1002/ece3.6839 (PMC7663068; doi:10.1002/ece3.6839)
Supplement: Supplementary file 1 — Appendix S1–S4 [file ECE3-10-12129-s001.docx]

| **Appendix table S1.** Accession numbers and vouchers for next generation sequencing. | | | | | | | | |  |
| --- | --- | --- | --- | --- | --- | --- | --- | --- | --- |
| Taxon | | GRIN accession # | | NCBI accession # | | Voucher accession # | | |  |
| *Medicago coronata* | | PI 498807 | | MT584353 | | I.S. Choi MD030 | | |  |
| *Medicago disciformis* | | PI 487327 | | MT584348 | | I.S. Choi MD032 | | |  |
| *Medicago tenoreana* | | PI 499160 | | MT584351 | | I.S. Choi MD027 | | |  |
| *Medicago arabica* | | PI 227031 | | MT584354 | | I.S. Choi MD031 | | |  |
| *Medicago sauvagei* | | PI 499155 | | MT584352 | | I.S. Choi MD028 | | |  |
| *Medicago secundiflora* | | PI 537239 | | MT584349 | | I.S. Choi MD026 | | |  |
| *Medicago praecox* | | PI 495418 | | MT584350 | | I.S. Choi MD033 | | |  |
| **Appendix table S2.** Accession numbers and vouchers for IR boundary inverstigation, obtained from USDA-GRNS. | | | | | | | | |  |
| Taxon | | GRIN accession # | |  | | Voucher accession # | | |  |
| *Medicago lupulina* | | PI 452459 | |  | | I.S. Choi MD034 | | |  |
| *Medicago lupulina* | | W6 24380 | |  | | I.S. Choi MD035 | | |  |
| *Medicago lupulina* | | PI 641645 | |  | | I.S. Choi MD036 | | |  |
| *Medicago lupulina* | | W6 16613 | |  | | I.S. Choi MD037 | | |  |
| *Medicago lupulina* | | PI 494656 | |  | | I.S. Choi MD038 | | |  |
| *Medicago lupulina* | | PI 269926 | |  | | I.S. Choi MD039 | | |  |
| *Medicago lupulina* | | PI 235103 | |  | | I.S. Choi MD040 | | |  |
| *Medicago lupulina* | | PI 202038 | |  | | I.S. Choi MD041 | | |  |
| *Medicago minima* | | PI 542796 | |  | | I.S. Choi MD042 | | |  |
| *Medicago minima* | | PI 538999 | |  | | I.S. Choi MD043 | | |  |
| *Medicago minima* | | PI 499047 | |  | | I.S. Choi MD044 | | |  |
| *Medicago minima* | | PI 499141 | |  | | I.S. Choi MD045 | | |  |
| *Medicago minima* | | PI 499109 | |  | | I.S. Choi MD046 | | |  |
| *Medicago minima* | | PI 499009 | |  | | I.S. Choi MD047 | | |  |
| *Medicago minima* | | PI 227372 | |  | | I.S. Choi MD048 | | |  |
| *Medicago minima* | | PI 226949 | |  | | I.S. Choi MD049 | | |  |
| *Medicago minima* | | W6 33640 | |  | | I.S. Choi MD050 | | |  |
| **Appendix table S3.** Information for populations and vouchers of *Medicago minima* and *M. lupulina*, collected from Texas, USA. | | | | | | | |  | |
|  | *Medicago lupulina* | | | | *Medicago minima* | | | | |
| Population | GPS coordinates | | Voucher # | | GPS coordinates | | Voucher # | plastome haplotype* (n) | |
| Bastrop 2019-18 | 29°55'10"N 97°24'17"W | | RCJ66 | | 29°55'10"N 97°24'17"W | | RCJ65 | O (10) | |
| Bastrop-Lee 2017-02 | 30°15’08”N 97°04’54”W | | RCJ73 | | N/A | | N/A | N/A | |
| Edwards 2018-11 | 29°42'51"N 100°20'21"W | | RCJ55 | | 29°44'41"N 100°23'40"W | | RCJ54 | O (8), A (2) | |
| Edwards 2018-12 | N/A | | N/A | | 29°29’23”N 100°09’46”W | | RCJ56 | O (10) | |
| Hays 2019-40 | 30°11’39”N 98°11’29”W | | RCJ97 | | 30°11’39”N 98°11’29”W | | RCJ96 | O (8), B (2) | |
| Kimble 2018-02 | N/A | | N/A | | 30°29'35"N 100°1'35"W | | RCJ111 | O (6), B (4) | |
| Real 2018-10 | 29°55'44"N 100°0'33"W | | RCJ43 | | 29°55'0"N 99°58'21"W | | RCJ42 | O (10) | |
| Robertson 2018-01 | 31°3'7"N 96°41'21"W | | RCJ84 | | 31°3'7"N 96°41'21"W | | RCJ83 | O (10) | |
| Robertson 2019-03 | 31°12'35"N 96°35'50"W | | RCJ89 | | 31°12'35"N 96°35'50"W | | RCJ88 | O (10) | |
| San Jacinto 2018-01 | 30°46'19"N 95°12'24"W | | RCJ99 | | N/A | | N/A | N/A | |
| Travis 2019-58 | 30°28’47”N 97°53’03”W | | RCJ29 | | 30°28’47”N 97°53’03”W | | RCJ28 | O (8), B (2) | |
| Travis 2019-59 | 30°35’12”N 97°57’23”W | | RCJ22 | | 30°35’12”N 97°57’23”W | | RCJ21 | O (8), B (2) | |
| Travis 2019-60 | N/A | | N/A | | 30°26’47”N 98°06’03”W | | RCJ15 | O (9), B (1) | |
| Travis 2019-61 | 30°22’39”N 98°03’38”W | | RCJ8 | | 30°22’39”N 98°03’38”W | | RCJ7 | O (9), B (1) | |
| Uvalde 2018-01 | 29°30'45"N 99°48'02"W | | RCJ39 | | 29°30'45"N 99°48'02"W | | RCJ38 | O (10) | |
| University of Texas at Austin | 30°17'14"N 97°44'19"W | | I.S. Choi MD078 | | 30°16'50"N 97°44'02"W | | I.S. Choi MD064 | O (10) | |
| Farwest (Austin TX) | 30°20'46"N 97°45'11"W | | I.S. Choi MD079 | | 30°20'46"N 97°45'11"W | | I.S. Choi MD065 | O (6), B (4) | |

**Appendix table S4.** Accession numbers for sequenced amplification products from *Medicago minima* and *M. lupulina*.

| Taxon | *Medicago lupulina* | | *Medicago minima* | | | |
| --- | --- | --- | --- | --- | --- | --- |
| Locus | Small IR_A_ | Small IR_B_ | J_LA_ | J_LB_ | J_SA_ | J_SB_ |
| NCBI accession number | MT832082 – MT832103 | MT832104 – MT832125 | MT846165 – MT846188 | MT846189- MT846212 | MT846213 – MT846389 | MT857027 – MT857203 |
